# The Effect of Daminozide, Dark/Light Schedule and Copper Sulphate in Tissue Culture of *Triticum timopheevii*

**DOI:** 10.3390/plants10122620

**Published:** 2021-11-29

**Authors:** Dmitry Miroshnichenko, Anna Klementyeva, Sergey Dolgov

**Affiliations:** 1 Kurchatov Genomics Center of All-Russia Research Institute of Agricultural Biotechnology, Timiryazevskaya Street 42, 127550 Moscow, Russia; 2 Branch of Shemyakin-Ovchinnikov Institute of Bioorganic Chemistry, Russion Academy of Science, 142290 Pushchino, Russia; anutik.vlasowa@yandex.ru (A.K.); dolgov@bibch.ru (S.D.); 3 All-Russia Research Institute of Agricultural Biotechnology, Timiryazevskaya Street 42, 127550 Moscow, Russia

**Keywords:** wheat, immature embryos, somatic embryogenesis, albino phenotype, 2,4-D, CuSO_4_

## Abstract

*Triticum timopheevii* Zhuk. is a tetraploid wheat that is utilized worldwide as a valuable breeding source for wheat improvement. Gene-based biotechnologies can contribute to this field; however, *T. timopheevii* exhibits recalcitrance and albinism in tissue cultures, making this species of little use for manipulation through genetic engineering and genome editing. This study tested various approaches to increasing in vitro somatic embryogenesis and plant regeneration, while reducing the portion of albinos in cultures derived from immature embryos (IEs) of *T. timopheevii*. They included (i) adjusting the balance between 2,4-D and daminozide in callus induction medium; (ii) cultivation using various darkness/illumination schedules; and (iii) inclusion of additional concentrations of copper ions in the tissue culture medium. We achieved a 2.5-fold increase in somatic embryogenesis (up to 80%) when 50 mg L^−1^ daminozide was included in the callus induction medium together with 3 mg L^−1^ 2,4-D. It was found that the dark cultivation for 20–30 days was superior in terms of achieving maximum culture efficiency; moreover, switching to light in under 2 weeks from culture initiation significantly increased the number of albino plants, suppressed somatic embryogenesis, and decreased the regeneration of green plants. Media containing higher levels of copper ions did not have a positive effect on the regeneration of green plants; contrarily, the elevated concentrations caused albinism in plantlets. The results and relevant conclusions of the present study might be valuable for establishing an improved protocol for the regeneration of green plants in tissue cultures of *T. timopheevii*.

## 1. Introduction

In recent decades, wheat species of the *Timopheevii* group with the G genome, such as *Triticum timopheevii* Zhuk. (2n = 4x = 28; A^t^A^t^GG) and its wild ancestor *T. araraticum* Jakubz., have received considerable attention as promising sources of immune genes. *T. timopheevii* is actively involved in modern breeding programs to create introgression lines and increase the genetic diversity of modern wheat due to its partial homology with the common wheat genome (BBA^u^A^u^DD) [1,2]. *T. timopheevii* has been shown to be a valuable donor for resistance genes to wheat fungal diseases [3,4,5], environmental stresses [6], and modified protein content [7]. In addition to direct crosses between Timopheev’s wheat and common wheat and the production of synthetic amphiploids, an acceleration of the wheat breeding process can be brought about by the application of the various biotechnological techniques. Genetic engineering and genome editing may serve as alternative means with which to study the functions of candidate genes and their regulatory elements, which control important traits of wheat with a G genome prior to introgression.

Genetic transformation and genome editing systems require several key components. The first important component is a regenerable target tissue into which the DNA, RNA, or RNP constructs can be introduced to generate modified plants with reasonable efficiency. Despite its great potential, Timopheev’s wheat has not received much attention for in vitro plant regeneration and genetic transformation. It displayed a low regeneration potential in both mature and immature embryo cultures [8]. Recently, moderate success was achieved in tissue cultures of *T. timopheevii* and artificially synthesized hexaploid *T. kiharae* (A^t^A^t^GGDD) [9]. This intermediate success allowed for the production of transgenic *T. timopheevii* plants, carrying the reporter gene *GFP* and the herbicide resistance gene *bar* for the first time [10]. However, the transformation efficiency was extremely low (0–0.5%) and only two independent transgenic plants were produced after biolistic mediated-delivery of the foreign DNA. The main obstacle for active use in genetic transformation experiments is the low regenerative capacity of *T. timopheevii* explants, coupled with the appearance of a high portion of albino plants [8,9]. Previously, the modification of the hormonal composition of the induction media increased the efficiency of somatic embryogenesis, but the high proportion of albino plants remained, especially at high auxin concentrations [9].

Albino plant formation is a well-known problem for androgenic cereal cultures, and it negatively affects the wider application of doubled haploid technology [11,12]. The frequency of chlorophyll-defective regenerants in anther cultures of *Triticeae,* including wheat, barley, rye, and triticale, can vary from almost all green to 100% albino plants [12]. The appearance of albino plants has also been reported in the embryo-derived cultures barley [13,14,15], switchgrass [16], sorghum [17], and rye [18]. The extent of albino plant formation is highly dependent on the genotype, and this influence is very difficult to overcome.

Albino plants lack chlorophyll pigment, they are unable to photosynthesize, and they eventually die outside the in vitro cultures. The precise mechanism underlying chlorophyll deficiency in regenerated plants has not yet been elucidated. It is associated with impaired chloroplast biogenesis and the inability of proplastids to differentiate into functional chloroplasts during the various phases of somatic embryo formation and development [19]. Since the process of chlorophyll biosynthesis requires a complex of elements and compounds, various approaches have been used to overcome albinism in tissue cultures with varying degrees of success. Appropriate external factors, such as phytohormones, chemical substances, and carbohydrates, have been actively examined in the context of albinism [11,12,20].

Plant growth regulators are the main inductors of morphogenic responses in cereals. Unlike androgenic cultures, where both cytokinins and auxins play an important role in plant regeneration, cultures derived from zygotic embryos are mainly stimulated by auxins, especially at the first stages, which are designed to induce caulogenesis. Exploration of various auxin types/concentrations in the embryo-derived cultures of G-genomic wheat showed that media initially supplemented with picloram provided a lower portion of chlorophyll-deficient plants than 2,4-D and Dicamba, especially in *T. kiharae* [9]. Moreover, it was hard to find a proper balance between the effective promotion of somatic embryogenesis and green plant regeneration in *Triticum timopheevii*, e.g., pilcoram was ineffective in stimulating somatic embryogenesis; dicamba allowed more plants to be generated, but a significant portion (up to 50%) were albino plantlets; 2,4-D induced more albino plantlets than picloram, although, due to the increased somatic embryogenesis efficiency, the total amount of regenerated green plants was higher as compared to other auxins [9]. The use of other plant growth regulators in conjunction with auxins may be useful to achieve a better balance between an efficient morphogenic response and the level of albino plant regeneration. Data from previous reports indicate that additional supplementation of cytokinin [21], an abscisic acid [22], a gibberellin inhibitor [23], or an ethylene inhibitor [24] positively affects the morphogenic response of embryo-derived cereal cultures, affecting the efficacy and the type of plant regeneration pathway.

Other changes in the formulation of the culture media, such as the type of carbohydrates, can also contribute to the progress in recalcitrant genotypes. It was previously reported that replacing sucrose with maltose as the carbon source has a positive effect on the green-to-albino-plants ratio and stimulated somatic embryogenesis in androgenic and embryo cultures of wheat and other cereals [25,26,27,28]. Indeed, in our previous study, the substitution of sucrose by maltose reduced albino plant formation in IE cultures of G-genomic amphiploid *T. kiharae*. In contrast, no significant difference in the number of albino plants was observed as a result of sugar type (sucrose, glucose, or maltose) in embryo cultures of *T. timopheevii,* while sucrose-containing media still tended to induce more green plants per explant than maltose, especially in *T. kiharae* [9].

Among the various attempts to reduce albinism, promising results have been obtained with the additional supplementation of copper ions to the medium. Copper ions are required for the conversation of amyloplasts into proplastids and chloroplasts and are involved in photosynthesis as a cofactor of plastocyanin, an essential electron carrier in the thylakoid lumen [29]. Copper ions are also known as active cofactors of several enzyme systems and Cu-containing proteins are involved in various biological processes [30]. Copper is an important >microelement of tissue culture media, which is usually supplied in the form of hydrated copper (CuSO_4_) in a concentration of 0.025 mg L^−1^ (0.1 µM) [31]. In the past years, the increased presence of copper sulphate (1 µM–80 µM) has been widely utilized to induce both somatic embryogenesis/plant regeneration and reduce the portion of albino plants in embryo-derived cultures and, especially, androgenic cultures of barley [32,33,34], sorghum [35], rice [36], wheat [37], and oat [38]. It was also reported that the presence of copper ions in tissue cultures affect the relationship between DNA methylation change and the number of regenerated green plants [39]. The effect of additional Cu supplementations, however, significantly depends on the genotype, since it was impossible to decrease the output of albino plants in certain genotypes of interest [38,39,40,41]. There is no information concerning the effect of additional CuSO_4_ concentrations in the tissue culture of G-genomic wheat, but such an approach should be investigated as a possible way to limit the formation of albino plants.

Effort should also be directed towards the modification of certain environmental factors, such as the duration of light/dark cultivation [42,43]. The general consensus from cereal researchers is that light is not obligatory for embryogenic callus induction. Conventional and widely practiced protocols include 3–5 weeks of culture of IEs under darkness with subsequent somatic embryo differentiation under illumination. The transfer to light, together with the removal of auxin, triggers the transition of proplastids to chloroplasts, and stimulates the process of maturation of somatic embryos and conversation into regenerants. Although most studies do not consider lighting requirements as critical for optimal somatic embryogenesis and cereal regeneration, the duration of the dark period and the timely transfer of the culture to illumination could be possible factors that influence the extent of albino plant formation in recalcitrant genotypes.

In the present study, we attempted to identify the strategy for increasing the efficiency of somatic embryogenesis and green plant regeneration in *T. timopheevii* cultures, while keeping the albino plant production at a low level. Various concentrations of daminozide, the inhibitor of gibberellin biosynthesis, were studied to increase the in vitro tissue culture efficiency of *T. timopheevii* IEs when supplied to the induction medium together with 2,4-D. The objective was also to determine the proper timing for transferring cultures from the dark to the light with regard to the efficacy of somatic embryogenesis and green/albino plants production. In addition, the possibility of decreasing the amount of chlorophyll-deficient plants was investigated by the supplementation of suitable doses of CuSO_4_ into media at the late stage of callus induction and the stage of pre-regeneration.

## 2. Results

### 2.1. Effect of Daminozide

**Figure 1:**
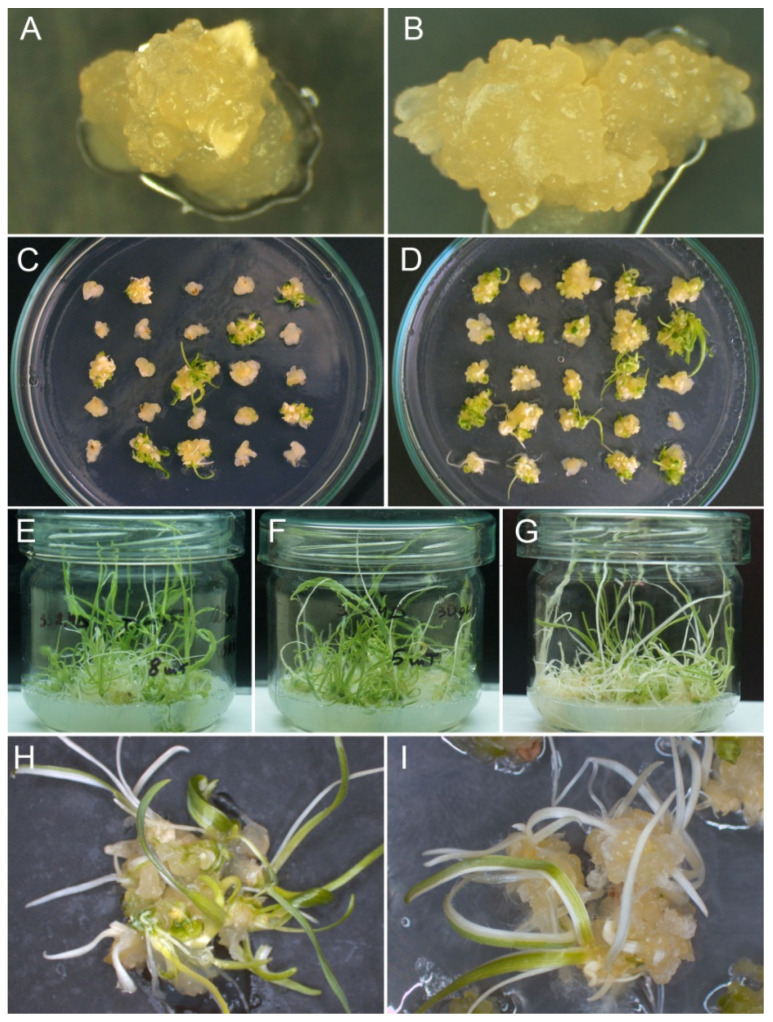
Somatic embryogenesis and plant regeneration in immature embryo-derived *Triticum timopheevii* cultures. The formation of the embryogenic callus on the induction medium supplemented with 3 mg L^−1^ 2,4-D (**A**) and 3 mg L^−1^ 2,4-D and 50 mg L^−1^ daminozide (**B**), after 25 days of culture. The formation of green buds and leafy structures after being transferred to the pre-regeneration medium from callus induced with 3 mg L^−1^ 2,4-D (**C**), 3 mg L^−1^ 2,4-D and 50 mg L^−1^ daminozide (**D**), after 40 days of culture. Regeneration of green/albino plantlets from callus induced using 3 mg L^−1^ 2,4-D (**E**), 3 mg L^−1^ 2,4-D and 50 mg L^−1^ daminozide (**F**), and 3 mg L^−1^ 2,4-D and 150 mg L^−1^ daminozide (**G**), after 65 days of culture. The occurrence of albino and green plantlets from the embryogenic callus exposed to the dark for 20 days (**H**) and 5 days (**I**) before being transferred to illumination, after 48 days of culture.

In this experiment, the effect of various daminodize concentrations on somatic embryogenesis and plant regeneration was assessed in combination with the auxin 2,4-D, which is commonly used for somatic embryogenesis in cereals. The proper concentrations of 2,4-D, which demonstrated better activity than other synthetic auxins, was established in a previous study [9]. The scutellum region of cultured *T. timopheevii* IEs began to swell within a week and the mass production of calli was observed after 2 weeks of culture. No differences in callogenesis among the tested daminozide concentrations were recorded, as all cultured IEs successfully produced calli. Within the third week of culture, the formation of the embryogenic callus was observed. In the IEs without daminozide, the lowest percentages of embryogenic callus formation were observed (Table 1), and the calli were more compact and contained fewer areas with morphogenic structures (Figure 1A) than in the IEs in presence of daminozide (Figure 1B). The statistical analysis (ANOVA) confirmed that the presence of daminodize had an effect on morphogenesis. Even at the lowest daminozide concentration, the rate of embryogenic callus formation was twice as high (60.9%) when compared with the medium supplemented only with auxin (31.0%). As the daminozide concentration was increased to 50–100 mg L^−1^, the rate of embryogenesis was enhanced; higher concentrations were less effective (Table 1). Most of the produced embryogenic calli were able to develop plants after transfer into the pre-regeneration medium (Figure 1C,D). The regeneration capacity fluctuated from 56.6% to 74.7% in the presence of daminozide, while without daminozide, it was approximately half, reaching 27.6% (Table 1).

Within 10–15 days of being transferred to the light, morphogenic calli formed both green and white sectors that started to convert into visible plantlets (Figure 1C,D). On average, 10–12 plantlets developed per single regenerating callus, and there was no significant difference between the studied concentrations (Table 1). Moreover, the ratio between the green and white plantlets was strictly dependent on the daminozide concentration (Figure 1E–G). The higher the concentration of daminozide applied, the higher the portion of albino plants observed. At a concentration of 150 mg L^−1^, each second plantlet was an albino (Table 1, Figure 1G), while on the medium without daminozide or that supplemented with the lowest daminozide concentration, approximately 75% of regenerated plants were green (Table 1, Figure 1E). On average, three albino plantlets were developed from embryogenic calli induced on the medium without daminozide and media supplemented with 12.5, 25, and 50 mg L^−1^ of daminozide (Table 1). At higher daminozide concentrations, the number of albino plants increased to five shoots per IE. The overall efficiency of green plant regeneration per initial IE was positively affected by daminozide (Table 1). The best efficiency was recorded at 50 mg L^−1^ of daminozide (Figure 1D,F). As a result of the good balance between the efficient embryogenic callus production (79.9%) and the moderate percentage of albino plantlets that formed (27%), the number of regenerated green plants per single initial IE was three times that of the daminozide-free medium (7.0 vs. 2.2). Moreover, regardless of the applied concentrations, the ratio and number of albino/green plants per single regenerating callus did not change.

### 2.2. Effect of Duration of the Dark/Light Cultivation

**Figure 2:**
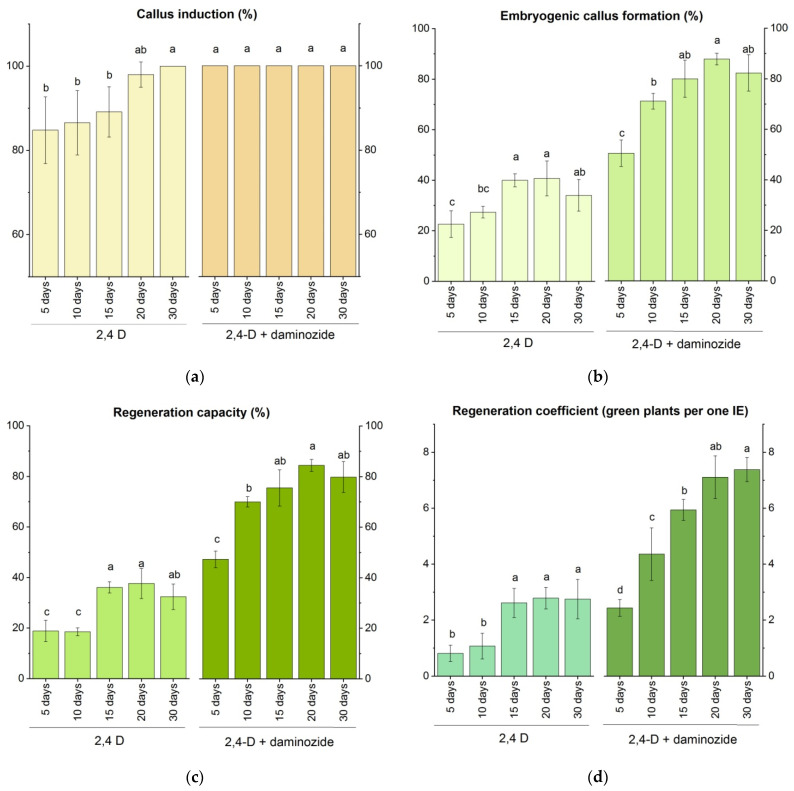
Effect of darkness/illumination regimes on the morphogenic response of *Triticum timopheevii* IEs cultivated on two callus induction media; the first was supplemented with 3 mg L^−1^ 2,4-D, the second was supplemented with 3 mg L^−1^ 2,4-D and 50 mg L^−1^ daminozide. Explants were cultivated in the darkness for 5, 10, 15, 20, and 30 days, and then were transferred to illumination (100 μmol m^–2^ s^–1^, with a photoperiod regime of 16 h light/8 h dark) for 25, 20, 15, 10, and 0 days, respectively, to achieve 30 days of culture on the induction medium. (**a**) The percentage of IEs that produced calli (%); (**b**) the percentage of IEs that produced embryogenic calli (%); (**c**) the percentage of regenerating calli (%); (**d**) the regeneration coefficient, calculated as the average number of green plants regenerated per single initial IE. Data collected from two media were separately subjected to one-way ANOVA; means with the same letter in the column had no significant differences according to Duncan’s multiple range test (*p* < 0.05).

**Figure 3:**
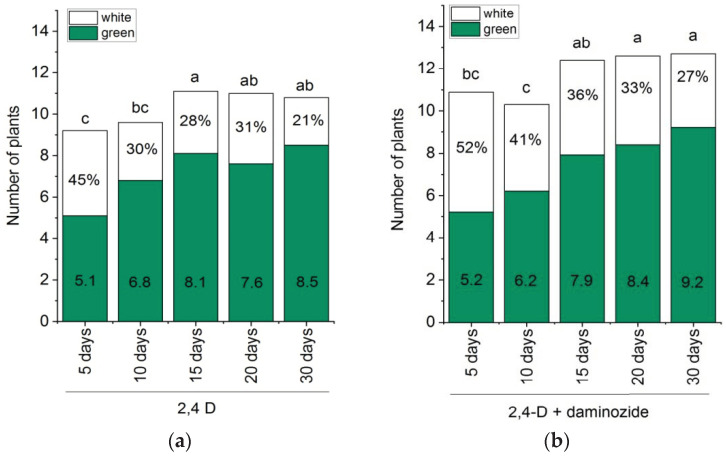
The regeneration response of embryogenic calli of *Triticum timopheevii* to the darkness/illumination regime and induction medium. Explants were cultivated in the darkness for 5, 10, 15, 20, and 30 days, and then were transferred to illumination (100 μmol m^–2^ s^–1^, with a photoperiod regime of 16 h light/8 h dark) for 25, 20, 15, 10, and 0 days, respectively, to achieve 30 days of culture on the callus induction medium. (**a**) IEs were cultivated on callus induction medium supplemented with 3 mg L^−1^ 2,4-D; (**b**) IEs were cultivated on callus induction medium supplemented with 3 mg L^−1^ 2,4-D and 50 mg L^−1^ daminozide. The stacked column is the average number of plants per regenerating callus and consists of mean numbers of albino plants (white box) and green plants (green box). The portion of albino plants (%) is indicated inside the white column; the mean number of regenerated green plants is indicated in the green box. Means with the same letter in the stacked column had no significant differences in the overall number of regenerated plants according to Duncan’s multiple range test (*p* < 0.05).

In this experiment, we modified the duration of the culture under darkness. Originally, the IEs were cultured for 30 days on the induction medium in the dark and then transferred on pre-regeneration and regeneration media to the light. In the present experiment, the dark culture duration was reduced in such a way that explants were initially exposed to the dark, and then, from the 5th, 10th, 15th, or 20th day after culture initiation, they were exposed to illumination. Two induction media were used: one medium was supplemented with 3 mg L^−1^ 2,4-D and 50 mg L^−1^ daminozide (the best variant from the previous experiment); the other variant was a medium that contained 2,4-D only. The effect of the duration of dark/light periods on embryogenesis and plant regeneration in response to cultivation on two media is shown in Figure 2 and Figure 3.

Analyses of variance indicated significant effects of shortened dark culture on both embryogenesis and plantlets production (*p* < 0.01). The reduced dark cultivation negatively affected the culture efficiency when using both of the tested induction media (Figure 2). On the medium supplemented with 2,4-D, the shorter the dark period, the lower the number of calli formed by the cultured IEs (Figure 2a). On both media, the dark period of 5–10 days was insufficient to stimulate the formation of embryogenic calli (Figure 2b); as a result, the percentage of regenerable calli also decreased (Figure 2c). The early transfer to the light increased the portion of albino plantlets to 45–52% and decreased the number of green plants to five plants per regenerable callus (Figure 3). The phenotype of albino plants ranged from completely white plants, plants with green and white sectors, to pale green plants (Figure 1H,I).

The prolongation of dark culture until 20 days significantly increased the number of embryogenic calli (Figure 2b), tended to reduce the portion of albino plantlets to 21–33% (Figure 3), and increased the number of regenerated green plants per IE (Figure 2d), especially on the medium with daminozide. As in the previous experiment, the medium with 2,4-D and daminozide was more effective than the medium supplemented with 2,4-D only when the same dark/light cultivation was compared. On both tested media, the highest rates of embryogenic and regenerable callus formation were found with the 20 days dark + 10 days light exposure. However, the LCD group did not confirm that this variance is statistically more efficient than the exposure to light after 15 or 30 days of culture (Figure 2b,c). Similarly, there were no significant differences in the ability of IEs to generate green plants in the case of cultivation in the dark for 1–15 days, 1–20 days, or 1–30 days, regardless of the medium tested (Figure 2d).

### 2.3. Effect of CuSO_4_ on the Generation of Green/Albino Plants

The experiment was designed to determine whether the additional Cu ion concentration in the medium favored the regeneration of green plantlets. The emerging embryogenic callus was exposed to a higher Cu level after 20 days of culture when the explants were transferred into the light on the fresh induction medium supplemented with various CuSO_4_ concentrations. The same CuSO_4_ concentrations were then also added to the pre-regeneration medium. Thus, the calli were subjected to a higher Cu level for 25 days, before transfer into the regeneration medium, which was free from additional CuSO_4_. Table 2 summarizes the effects of the elevated copper content in the culture of *T. timopheevii* IEs.

In general, no clear positive effect related to additional CuSO_4_ concentrations was found. According to Fisher’s protected LSD means comparison procedures, the values of somatic embryogenesis and regeneration did not differ significantly from the control at *p* < 0.05 (Table 2). The efficacy of embryogenic and regenerable callus formation fluctuated in the range 81–89% and 78–88%, respectively, which was similar to that observed on the standard MS medium (82.5%). It was evident that high CuSO_4_ concentrations had a toxic effect, as the supplementation of 50 and 100 µm copper sulphate suppressed the formation of embryogenic calli, negatively affected regeneration, and increased the portion of albino plants (Table 2). Surprisingly, all tested CuSO_4_ concentrations tended to increase the number of albino plantlets, since the portion of albino shoots was generally higher (32–44%) as compared to the medium without additional copper (28%). The overall number of regenerated green/albino plants per regenerable calli increased moderately when calli were cultivated in the presence of 15–25 µm CuSO_4_, reaching 14–15 shoots per callus. The observed increase, however, did not affect the overall green plant generation. LSD-based multiple comparisons showed that the regeneration coefficient remained almost unchanged, fluctuating in a range of 6.8–7.5 shoots per IE, and was similar to that of the medium without additional copper sulfate (7.3 shoots per IE).

## 3. Discussion

The experiments carried out in the present study demonstrated the strong recalcitrance of the *T. timopheevii* IE cultures, especially with regard to green/albino plant regeneration. Herein, we achieved a substantial increase in the number of embryogenic and regenerable calli in *T. timopheevii* IE cultures of up to 2.5 times as a result of the incorporation of daminozide into the induction medium. The joint action of daminozide and auxin 2,4-D significantly increased the proportion of morphogenic structures (Figure 1B,D). This result is consistent with the previous study in recalcitrant einkorn wheat that demonstrated that daminozide in combination with various auxin-like substances can modify the cell differentiation pathway, promoting the formation of numerous nodular structures, and resulting in an increase in the percentage of morphogenic explants from 8% to 52% [21]. Daminozide is well known as an anti-gibberellin compound. Its mode of action includes blocking 3β-hydroxylation from GA_20_ to GA_1_ [44]. Since daminozide inhibits the late stages of GA biosynthesis, it may also induce various side biosynthesis pathways [45], thus exerting various direct and non-direct actions in tissue cultures. In plants, it was reported to cause a significant reduction in the total antioxidant capacity [46]. Daminozide also inhibited ethylene production in fruits by blocking the conversion of methionine to aminocyclopropane-1-carboxylic acid [47]. In human cells, daminozide was shown to inhibit 2-oxoglutarate (2OG) oxygenases by chelating the active site metal via its hydrazide carbonyl and dimethylamino groups [48]. There is a supposition that daminozide in combination with auxin can act as a stress inducer in tissue cultures, contributing to morphogenesis; this can occur through the redistribution of nutrients [21]. Histological observation showed that the addition of daminozide to callus induction medium stimulated the accumulation of various storage preserves in the embryogenic structures of einkorn [21]. This correlates with the finding that the application of this substance induced the allocation of photoassimilates and dry matter into the leaves in leafy plants [45,46]. Although daminozide’s exact mode of action remains elusive, the positive effect of daminozide on embryogenic tissue initiation in einkorn [21], loblolly pine [49], and Timopheev’s wheat suggests its practical potential for use in the tissue culture of various species, especially cereals.

Moreover, the number of plantlets per regenerating calli in *T. timopheevii* did not significantly change after supplementation of daminozide and was in the same range of 10–12 plantlets as in the control variant. This observation indicated that the stages of tissue culture after callus initiation need to be improved in such a way that the multiple induced morphogenic structures are able to develop into plantlets. Our previous attempts to modify pre-regeneration and regeneration media composition by adding various cytokinins or substituting carbohydrates were unsuccessful in promoting better regeneration abilities in embryogenic *T. timopheevii* calli [9].

The modification of the dark/light conditions during the callus induction medium stage assessed in the present study did not provide positive evidence for the possibility of increasing the number of generated plants in *T. timopheevii*. Moreover, it became obvious that reducing the dark cultivation period to 5–10 days negatively affects the ability of IES to produce morphogenic calli, especially without the addition of daminozide. For the successful formation of embryogenic callus, the *T. timopheevii* IEs should be cultivated in the dark for at least 2 weeks after culture initiation. Our observation is consistent with the dark/light experiments conducted on a thin cell layer culture of apical meristematic tissue of rice, which revealed the importance of 2 weeks of exposure to darkness for shoot regeneration before exposure to light [50]. Moreover, in mature rice embryo cultures, it was reported that illumination should be excluded or reduced at the beginning of the regeneration stage, because extending the dark treatment for 1 week improved the percentage of calli with green areas as compared to direct or diffuse light groups, although the regeneration efficiency was not significantly affected [42]. In accordance with this, low-intensity light (20 µmol m^−2^ s^−1^, 16 h photoperiod) at the pre-regeneration stage substantially improved the regeneration frequency of winter wheat calli produced from IEs, as compared with cultivation under constant high-intensity illumination (50 µmol m^−2^ s^−1^) [43].

In anther bread wheat cultures, the continuous dark application during the callus induction stage better stimulated callus formation than continuous light or ‘15th to last day light’ regimes [51], which is similar to our observations. Moreover, the authors reported that continuous dark and continuous light regimes were near equal in promoting plant regeneration and even outperformed the ‘15th to last day light’ variant [51]. Another study dealing with wheat haploid production showed that combined light/dark cultivation of explants (12 h photoperiod) was not significantly better for somatic embryo production, plant regeneration, or frequency of green plants as compared with a continuous dark culture [52]. Exposure to continuous high-intensity light was found to positively affect cell differentiation, as each IE-derived callus was able to produce green spots and leafy structures [43]. We assumed that early exposure to illumination in our experiments (80 µmol m^−2^ s^−1^) would help reduce the number of albino plants. This assumption was not confirmed, since the earlier the callusing *T. timopheevii* explants were transferred to the light, the greater the number of white shoots developed, regardless of the hormonal composition of the medium. It should be noted that the ‘continuous light’ condition was not used here and explants were subjected to a 16 h photoperiod, so it is problematic to make direct comparisons with the aforementioned publications.

The previously reported efforts directed towards reducing albinism demonstrated that incorporation of additional copper in the cultivation medium may significantly reduce the output of chlorophyll-deficient regenerants. A higher level of copper sulphate (5–100-fold higher as compared to the MS medium) was found to improve the efficiency of somatic embryogenesis and green plant regeneration in IE-derived cultures of barley [13,53,54], bread wheat [55], indica rice [36], and sorghum [35]; however, the optimal Cu^2+^ content depended on the genotype. A higher level of copper ions was also successfully used to reduce the regeneration of albino plants in androgenic cultures; moreover, it became possible to produce green plants from cultivars that previously generated only albino plantlets [33,56]. In the present study, various concentrations of CuSO_4_ (5–100 µM) were analyzed, but none demonstrated a positive effect. Surprisingly, supplementation with additional Cu2+ increased the albino/green plants ratio in our study, as the number of chlorophyll-deficient plantlets tended to increase at all evaluated concentrations. Our data contradict the aforementioned studies, but the observed discrepancy is not exceptional. Recently, Malik et al. (2021) [24] reported that the calli of mature wheat embryos exposed to CuSO_4_ concentrations in excess of the MS control medium showed a decrease in the frequency of somatic embryogenesis and plant regeneration. The addition of copper did not confirm the possibility of increasing regeneration in oat androgenic cultures [38] and reducing albinism in the barley microspore cultures [38].

Many efforts have been directed at reducing the portion of albinos among regenerated plants. Taking into account the results of our previous studies [8,9], we can assert that *T. timopheevii* is a very recalcitrant wheat species, as changing the plant phytohormones, modifying the carbohydrates content, varying the dark/light schedules, and supplementation with additional copper ions did not positively influence the albino/green plants ratio. We suppose that this phenomenon is characteristic of G-genomic wheat species. The appearance of albino plants was also previously observed in IE cultures of the hexaploid *T. kiharae* (A^t^A^t^BBGG genome) [9], another member of *Timopheevii* group. *T. araraticum* Jakubz., the non-cultivated wild species that possesses the same A^t^A^t^GG genomic composition as *T. timopheevii*, also exhibited a significant portion of albinos in IE cultures. In our preliminary experiment (according to the protocol described here), each second embryogenic *T. araraticum* calli generated albino plants and the average ratio of albino to green plants fluctuated around 1:5 (data in preparation). Moreover, the formation of albino plants in zygotic embryo cultures of wheat species/cultivars with the genomic composition A^u^A^u^BB or A^u^A^u^BBDD is predominately sporadic and does not interfere with further practical applications [8,57]. The impact of genotype on the occurrence of albino plants is more prominent in androgenic cultures of cereals. Albinism in anther cereal cultures was accompanied by various changes that take place in the chloroplast genome, such as the micro- and macro-deletion of photosynthetic and structural plastid-localized genes [58,59] and changes in their copy number [19]. On the other hand, the appearance of the albino phenotype is also controlled by various nuclear-localized genes, without changes in the plastid genome [60]. To date, various nuclear-encoded factors, such as QTLs associated with albino or green plant regeneration in androgenic cultures, have been mapped in cereals [20,61,62]. Along with this, the impact of differently expressed nuclear genes and proteins has also been documented [62,63].

In barley, it was found that the ability of genotypes to regenerate green plants in androgenic cultures depends on the level of differentiation of microspore plastids in vivo [19]. It has been suggested that the molecular changes leading to the albino phenotype are genetically determined before the introduction of microspores into the in vitro culture [64]. This means that the modification of the tissue culture procedure cannot reverse the differentiation of proplastid into amyloplast and help reduce albinism in vitro. Although there is no information concerning plastid biogenesis during zygotic embryogenesis in *T. timopheevii*, our data indicate that the G genome is probably a stronger determinant than the various endogenous factors for the occurrence of the albino phenotype in IE cultures. Taking into account the hypothesis mentioned above [64], further research should be directed towards the adjustment of conditions before an in vitro culture initiation. For example, chemical or thermal pre-treatment of the freshly isolated embryos or even in vivo stressing of the young spikes could be applied to interrupt the nuclear/plastid genome interactions involved in chlorophyll-deficient plant regeneration in the *T. timopheevii*.

Therefore, it can be concluded that various modifications, such as the increased level of copper ions in the medium and shifting the dark/light cultivation durations, did not improve the culture efficiency of IE-derived cultures of *T. timopheevii*. We found that there is no need to increase the concentration of copper sulphate since it negatively affected the green-to-albino-plants ratio. Our data also indicate that the transfer of dark-induced calli to illumination in under 2 weeks from the initiation of the culture is prohibited since it significantly reduces somatic embryogenesis and increases the albinism of regenerated plants. The substantial increase in the efficiency of somatic embryogenesis was achieved in IE *T. timopheevii* cultures due to the inclusion of daminozide into the auxin-containing induction medium. This approach can be further used for recalcitrant species and wheat cultivars to increase the culture efficiency and develop improved protocols for plant regeneration and genetic transformation.

## 4. Materials and Methods

### 4.1. Plant Material

For isolation of immature zygotic embryos (IEs), the donor plants of Timophefeev’s wheat (*T. timopheevii* (Zhuk.), accession number K-47793) were grown in a glasshouse with a photoperiod of 16 h/8 h (day/night) at 25 °C ± 2/20 °C ± 2 day/night temperature. IEs ranging from 1 to 2 mm in length were freshly isolated according to the method used by Miroshnichenko et al. (2016) [9] and were used as explants in all experiments.

### 4.2. Culture Media and Culture Methods

All culture media used in experiments contained MS salts and vitamins [31] and were solidified with agar (European type technical grade, Panreac, Spain) concentrated at 0.7% (*w*/*v*). The callus induction medium was supplemented with 30 g L^−1^ sucrose, 150 mg L^−1^ asparagine, and 3 mg L^−1^ 2,4-D. Succinic mono-N,N-dimethylhydrazide (daminozide) was added to the callus induction media at six concentrations: 0, 12.5, 25, 50, 100, and 150 mg L^−1^. For one experiment, a Petri dish with 25 immature embryos was used per daminozide treatment. The experiment was repeated six times. Isolated IEs were cultured in the dark for 30 days at 24 ± 2°. Then, all calli developed from IEs were transferred onto the pre-regeneration medium (phytohormone-free MS medium supplemented with 30 g L^−1^ sucrose) and incubated for 15 days in Petri dishes at 24 ± 2 °C under light (100 μmol m^–2^ s^–1^) with a photoperiod regime of 16 h light/8 h dark. Thereafter, the morphogenic calli were transferred into culture flasks, with 8–10 morphogenic calli each, containing the phytohormone-free MS medium supplemented with 20 g L^−1^ sucrose (regeneration medium), and cultured for 30 days at 24 ± 2 °C under light (100 μmol m^–2^ s^–1^ provided by Philips cool white and OSRAM fluora fluorescent lamps) with a photoperiod regime of 16 h light/8 h dark.

To study the effect of the dark/light regime on the albino/green plant formation, two callus induction media were selected. The explants were cultured on medium supplemented with 3 mg L^−1^ 2,4-D or on the medium supplemented with 3 mg L^−1^ 2,4-D and 50 mg L^−1^ daminozide. In all variants, explants were cultured on the callus induction medium for 30 days. In one variant, the isolated embryos were cultured solely in the dark for 30 days. In other variants, the dark cultivation period was reduced. Petri dishes were transferred to the light after 5, 10, 15, and 20 days of dark culture, and exposed to illumination (~100 μmol m^–2^ s^–1^) with a photoperiod regime of 16 h light/8 h dark. The produced calli were transferred onto the pre-regeneration medium (described in the previous experiment) for 15 days and then regenerating calli were cultured for 30 days on the regeneration medium (described in the previous experiment). At all stages of the experiment (induction, pre-regeneration, regeneration), the cultures were incubated in a culture chamber at 24 ± 2 °C and the same light regime (~100 μmol m^–2^ s^–1^ from an equal mix of Philips cool white and OSRAM fluora fluorescent lamps for a 16 h per day photoperiod) was used. For one experiment, a Petri dish with 25 IEs was used per dark–light treatment/hormonal composition; the experiment was repeated five times.

Various concentrations of CuSO_4_ were investigated to increase the portion of green plants. In this experiment, IEs were cultured in the dark for 20 days on the callus induction media supplemented with 3 mg L^−1^ 2,4-D and 50 mg L^−1^ daminozide; then, explants were transferred to the light for 10 days onto the same callus induction medium but supplemented with 0, 5, 10, 15, 25, 50, 100 µM CuSO_4_. Thereafter, all induced calli were transferred onto the phytohormone-free pre-regeneration medium supplemented with the corresponding concentration of CuSO_4_ and cultured for 15 days under light in Petri dishes. The morphogenic calli were then transferred into culture flasks containing the phytohormone-free medium without CuSO_4_. At all stages of experiment (induction, pre-regeneration, regeneration), the cultures were incubated in a culture chamber at 24 ± 2 °C and the same light regime (~100 μmol m^–2^ s^–1^ from equal mix of Philips cool white and OSRAM fluora fluorescent lamps for a 16 h per day photoperiod) was used. For one experiment, a Petri dish with 20 IEs was used per copper concentration; all treatments had four replicates.

### 4.3. Statistical Analysi

Data were collected on a per plate/jar basis and each Petri plate or jar was considered to be an experimental unit. Identical experiments were carried out at different times to verify the reproducibility of the results. For each replication, IEs were isolated from spikes of a new set of greenhouse-grown plants. The rate of embryogenic callus formation and the percentage of regenerating calli were calculated per initial number of IEs after 45 days of culture. The percentage of regenerating calli was expressed as percentages of IEs that produced calli with at least one green/albino developing plantlet longer than 1 cm. The mean number of shoots per regenerating callus was estimated by counting both green and albino plantlets; the number of albino plants and the percentage of albino plant formation were also counted. The regeneration coefficient was calculated as an average number of regenerated green plants per single initial IE. The statistical analysis was conducted by means of Statistica10 software (©StatSoft Inc, Tulsa, OK, USA). Data were subjected to one-way analysis of variance (ANOVA) followed by mean separation by LSD test at *p* ≤ 0.05.

## Figures and Tables

**Table 1:** The effect of different concentrations of daminozide added to the callus induction medium containing 3 mg L^−1^ 2,4-D on somatic embryogenesis and plant regeneration of *Triticum timopheevii* IEs.

Daminozide Concentration (mg L^−1^)	Embryogenic Callus Formation (%)	The Percentage of Regenerating Calli (%)	No. of Plantlets per Regenerable Calli				Regeneration Coefficient *
			Total	Green Plants	Albino Plantlets	Albino Portion (%)	
0	31.00 a	27.6 a	11.0 ab	8.2 b	2.8 a	25	2.6 a
12.5	60.9 bc	56.6 bc	10.2 ab	7.7 b	2.5 a	24	4.1 ab
25	68.6 bcd	63.5 bcd	11.7 ab	8.7 b	3.0 a	26	6.0 bd
50	79.9 d	74.7 d	12.0 ab	8.8 b	3.3 a	27	7.0 d
100	72.7 cd	69.8 cd	12.2 b	7.1 ab	5.1 b	42	5.1 b
150	58.4 b	57.5 b	9.9 a	4.9 a	5.0 b	50	2.0 a

* average number of green plants regenerated per single IE. Calli were initiated from IEs within 30 days on callus induction medium supplemented with 3 mg L^−1^ 2,4-D with the subsequent subcultivations on a medium without phytohormones. Means with the same letter in the column had no significant differences according to Duncan’s multiple range test (*p* < 0.05).

**Table 2:** The effect of additional CuSO_4_ concentrations on somatic embryogenesis and plant regeneration from calli derived from *Triticum timopheevii* IEs.

Additional CuSO_4_ µM *	Embryogenic Callus Formation (%)	The Percentage of Regenerating Calli (%)	No. of Plantlets per Regenerable Calli				Regeneration Coefficient **
			Total	Green Plants	Albino Plantlets	Albino Portion (%)	
0	82.5 c	82.5 b	12.5 a	9.0 b	3.5 a	28	7.3 b
5	83.8 c	82.5 b	12.8 ab	8.2 ab	4.6 ab	36	6.8 b
10	88.8 c	85.5 b	13.7 ab	8.5 ab	5.2 ab	38	7.3 b
15	82.5 c	77.5 b	14.0 ab	9.4 b	4.7 ab	33	7.3 b
25	81.1 c	81.0 b	14.5 b	9.2 b	5.3 b	37	7.4 b
50	67.6 b	59.5 a	13.3 ab	7.9 ab	5.4 b	41	4.7 a
100	56.0 a	45.0 a	11.9 a	6.7 a	5.2 ab	44	3.2 a

* The standard MS medium contained the 0.1 µm CuSO_4_; ** Average number of green plants regenerated per single initial IE. Calli were initiated from IEs within 20 days on callus induction medium supplemented with 3 mg L^−1^ 2,4-D and 50 mg L^−1^ daminozide in the dark, with the subsequent subcultivation on the same callus induction medium supplemented with additional CuSO_4_ concentrations under illumination (100 μmol m^–2^ s^–1^, with a photoperiod regime of 16 h light/8 h dark) for 10 days, and then transferred for 15 days to illumination on the pre-regeneration medium without plant growth regulators containing the same CuSO_4_ concentration. Means with the same letter in the column had no significant differences according to Duncan’s multiple range test (*p* < 0.05).

## Data Availability

The data generated or analyzed during this study are included in this published article. The datasets used and analyzed during the current study are available from the corresponding author on reasonable request.
